# The effect of disclosure on adherence to antiretroviral therapy among adults living with HIV in Ethiopia: a systematic review and meta-analysis

**DOI:** 10.1186/s12879-019-4148-3

**Published:** 2019-06-17

**Authors:** Getenet Dessie, Fasil Wagnew, Henok Mulugeta, Dessalegn Amare, Dube Jara, Cheru Tesema Leshargie, Ayenew Negesse, Swati Rayasam, Sahai Burrowes

**Affiliations:** 1 Department of Nursing, school of health science, College of medicine and Health Science Bahr Dar University, P.O. Box 79, Bahir Dar, Ethiopia; 2 Department of Nursing, College of Health Science Debre Markos University, P.O. Box 269, Debre Markos, Ethiopia; 3 Department of Public Health, College of Health Science Debre Markos University, P.O. Box 269, Debre Markos, Ethiopia; 4 Department of Environmental Health, College of Health Science Debre Markos University, P.O. Box 269, Debre Markos, Ethiopia; 5 Department of Human Nutrition and Food Sciences, College of Health Science Debre Markos University, P.O. Box 269, Debre Markos, Ethiopia; 6 Independent Researcher, Berkeley, CA USA; 7 Public Health Program, College of Education and Health Sciences, Touro University California, Vallejo, USA

**Keywords:** Adherence, Antiretroviral therapy, HIV/AIDS, Disclosure, Ethiopia

## Abstract

**Background:**

Several factors have been identified as being associated with increased adherence to antiretroviral therapy, including sero-status disclosure; however, studies examining the effect of disclosure on ART adherence in Ethiopia have had inconsistent findings. This systematic review and meta-analysis therefore aims to estimate the pooled effect of disclosure on adherence to ART among adults living with HIV in Ethiopia.

**Methods:**

We performed a systematic search for articles reporting on peer-reviewed, quantitative, English-language observational studies of reporting the association between self sero-status disclosure and good ART adherence in adults living with HIV/AIDS in Ethiopia during published from 2010 to 2015. We searched four electronic databases: PubMed/Medline, the World Health Organization’s Hinari portal (which includes the SCOPUS, African Index Medicus, and African Journals Online databases) for studies from December 1, 2017 to January 30, 2018. We also searched university repositories and conference abstracts for unpublished studies. We conducted a meta-analysis for the pooled effect of adherence using a random effects model in Stata version 14 and assessed publication bias using the Egger’s test for funnel plot asymmetry.

**Results:**

Our search returned in 179 studies, of which seven (3.9%), were eligible and included in the final meta-analysis. The seven included studies were conducted from 2010 to 2015. Our analysis found that disclosure had a significant effect on the adherence to ART in adult patients living with HIV. Patients who disclosed were 1.64 times more likely to have good adherence to ART compared with those who did not (OR: 1.64, 95% CI: 1.11, 2.42). The small number of studies eligible for review and differences in study definitions of adherence and disclosure were the main limitations of this study.

**Conclusion:**

This review found a statistically significant positive effect of disclosure status on the adherence to ART in adult patients living with HIV in Ethiopia. This suggests that Ethiopia’s national treatment and prevention programs should redouble efforts to encourage self-disclosure among people living with HIV/AIDS. Encouraging supportive social environments for disclosure, and promoting partner notification and partner disclosure support initiatives might be particularly helpful in this regard.

## Background

The unprecedented global response to the HIV/AIDS pandemic of the 1990s brought millions of people living with HIV/AIDS (PLWHA) access to lifesaving antiretroviral therapy (ART). The success of these programs and the continuing challenges in developing and implementing effective HIV prevention interventions have meant that the number of people receiving ART has continued to grow steadily. It is estimated that 21.7 million people worldwide were receiving ART by the end of 2017 [1]. In the region of the world most heavily affected by HIV/AIDS—eastern and southern Africa—UNAIDS estimates that 65% of PLWHA are receiving ART [2]; in Ethiopia, 75% of adult PLWHA (approximately 415,578 people) were receiving ART in 2017 [2]. While the growth of ART treatment programs in sub-Saharan Africa is impressive, these programs are fragile: heavily reliant on a few, low-cost ART drug regimens primarily financed by external donors [3]. High levels of patient adherence to ART (e.g., 95% of doses taken), crucial for maintaining the health of PLWHA, have become even more important in this context of limited antiretroviral drug choice, as adherence is crucial for preventing resistance to these drugs. Unfortunately, maintaining good patient adherence to ART in these programs continues to be challenging.

Studies in low-income countries have identified several factors that affect adherence to ART, of which disclosure of patient sero-status is a major contributing factor [4–7]. Social support, access to mental health care, counseling, educational interventions, and good nutrition have also been found to be associated with increased adherence to ART [4–7]. Treatment factors such as the complexity of the drug regimen and provider factors such as the quality of relationships between providers and patients have also been found to influence adherence rates [8–10].

The disclosure of one’s HIV status to relatives, friends, and sexual partners has significant health implications. Studies have found that patients who disclosed their sero-status had better social support; stronger family and relationship cohesion; reductions in anxiety and depression; improvements in physical health, emotional support, and financial support; and were better able to take ART freely and to improve their ART adherence [11–13]. Conversely, high levels of employment discrimination, fear of being abandoned by family members, fear of divorce, and communication difficulties were the most important barriers to disclosing sero-status [11, 14–16].

While the relationship between disclosure and adherence is fairly well-established in high-income settings, fewer studies have investigated the association in sub-Saharan Africa. Studies that have examined the association between ART adherence and disclosure among PLWHA in Ethiopia in particular have presented inconsistent results, with some finding strong positive associations and others finding limited or even negative associations between the two factors [17, 18]. The one existing systematic review of factors related to adherence in sub-Saharan Africa included only a handful of articles that examined the effect of disclosure specifically [6].

This systematic review and meta-analysis seeks to fill the gaps in the Ethiopian literature by estimating the pooled effect of disclosure status on adherence to ART among adult patients living with HIV in Ethiopia. Understanding Ethiopia’s specific adherence challenges is important, as the country has a large national ART treatment program serving extremely heterogeneous geographic regions and socio-economic groups. Although the country’s overall adult HIV prevalence has declined significantly in the last two decades [19], the epidemic is spread among the adult population with large geographic and sub-population variation in prevalence and important regional hotspots with very high prevalence occurring in large urban areas and certain regions such as Gambella [20]. Determining whether the impact of disclosure on adherence is as strong in Ethiopia as in other settings, or whether is there are regional or sub-population variation in the importance of disclosure might, therefore, be useful for policy makers trying to determine the amount of resources to dedicate to disclosure promotion activities. Being efficient in HIV program resource allocation is particularly important as Ethiopia continues to expand its national program to rural areas while shifting donor priorities and institutional changes [3, 21]. We hope that our findings will be useful to these stakeholders who are interested in designing appropriate interventions to improve ART adherence and sero-status disclosure among PLWHA.

## Methods

We conducted this systematic review and meta-analysis per PRISMA guidelines [22]. This study was not pre-registered.

### Eligibility criteria, data sources and search strategy

We performed our electronic database search using PubMed/MEDLINE, Hinari (a World Health Organization sponsored database that provides low-income researchers access to the SCOPUS, African Index Medicus, and African Journals Online databases), Google Scholar, and the Cochrane Library.

We built a search strategy by using the Boolean operator “and/or” with combinations of keywords. For example, (“adherence” AND “antiretroviral” AND “association” OR “determinant” AND “disclosure” AND “therapy” AND “Ethiopia”). For PubMed’s advance search, we used Medical Subject Headings (MeSH terms, See Appendix) to expand the search strategy. Unpublished studies were also accessed through the Addis Ababa University library. The reference lists of included studies were also reviewed to retrieve additional studies. The Cochrane library was explored in an effort to confirm whether there were existing systematic reviews or meta-analyses and to check the availability of ongoing projects related to the current systematic review and meta-analysis.

Eligible publications were peer reviewed, English-language scholarly articles or PhD dissertations, and peer reviewed conference abstracts published between the years 2010 to 2015; for observational, quantitative studies that took place in Ethiopia; and that investigated the association between sero-status disclosure and adherence to ART among adults (age 18 years and greater) living with HIV and currently receiving antiretroviral therapy. We excluded the studies focusing on children, review articles, and articles that had methodological problems after being reviewed by two authors using the Newcastle-Ottawa Scale (NOS) criteria [23].

### Data abstraction and quality assessment

Two reviewers screened the abstracts and titles of articles found by the search for relevance and for match with our inclusion criteria. When it was unclear whether an abstract was relevant or not, it was included for retrieval. The two reviewers then retrieved the full text of the screened articles then assessed them for relevance based on their topic, study outcomes, and methodology. Articles deemed irrelevant to the study were removed at this stage and the full text of the remaining articles reviewed for quality, by two study investigators using the Newcastle-Ottawa Scale (NOS). Articles whose NOS quality scores were less than an average score of six by the two investigators were excluded from the final analysis. Discrepancies were resolved with a third reviewer whenever appropriate.

### Data analysis and synthesis

Data were extracted from each of the original studies using Microsoft Excel and Stata version 14 for further analysis. Heterogeneity was checked by using an I^2^ test statistic [24]. We used a forest plot to visualize the presence of heterogeneity. Since there was relatively moderate heterogeneity, we used a random effects model for analysis to estimate the pooled effect. We also used funnel plot asymmetry and Egger’s test of the intercept to check for publication bias. [25]. To verify the results, two researchers independently computed the main statistical analyses and checked for consistency. The effect size estimates were converted to odds ratios, as all included studies compared two groups and reported dichotomous outcomes.

## Results

### Selection and identification of original studies

**Fig. 1:**
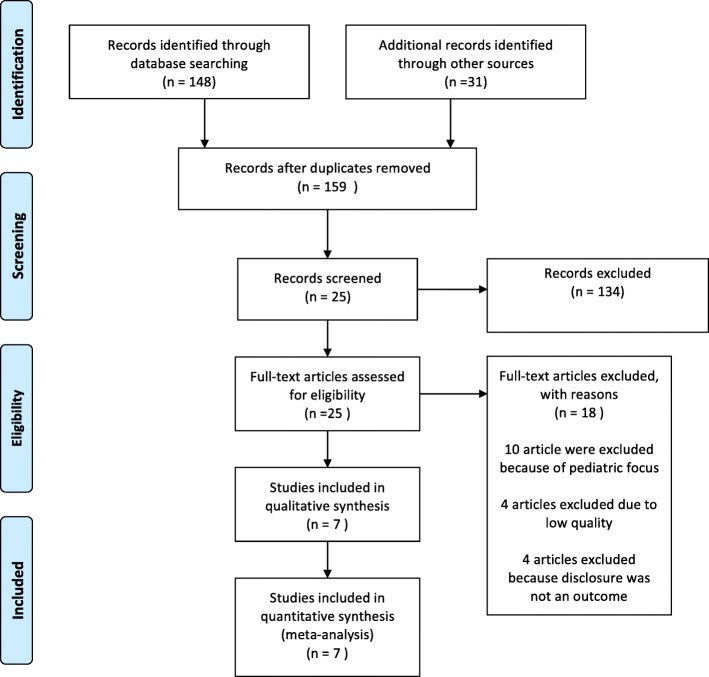
PRISMA Flow diagram showing the procedure of selecting studies for meta-analysis, 2010–2015, Ethiopia

Our search identified 179 published articles, of which 148 articles were found in the PubMed, Hinari and Scholar databases; the remaining were found in university libraries, conference abstracts, or reference lists (see Fig. 1). Of the total identified, 154 articles were excluded at initial assessment after reviewing their titles based on the inclusion criteria. The abstracts and full text of the remaining 25 studies were assessed and screened for eligibility criteria and whether they reported the outcome variable of interest (adherence); 18 articles were excluded due to failure to meet inclusion criteria. Two articles were excluded due to poor quality based on the selection criteria [17, 26]. Four articles were excluded because their regression tables did not include disclosure as an independent variable [27–30]. Two articles were excluded because their results did not report odds ratios or statistics that could be converted into odds ratios [31, 32]. Seven studies met the eligibility and quality criteria and were included in the analysis.

### Study measures

For the purpose of this review, we allowed the broadest definition of “disclosure” employed by the studies under review, defining it as disclosure to community members (inclusive of family, religious leaders, friends etc.). We also the broadest definition of “good adherence” employed by the studies under review: defining it as fewer than three missed doses per day over the study period. Table 1 below lists how each of the seven included studies defined disclosure and good adherence. Our principal summary measure was odds of “good adherence”, i.e., an odds ratio either directly reported or calculated from the study outcomes presented.

**Table 1:** Characteristics of included studies for meta-analysis, 2010–2015, Ethiopia

Study #	Author	Publication year	Region	Sample size	Good Adherence Definition	Disclosure Outcomes	Good adherence		OR
							Among disclose	Among non-disclose	
1	Mitiku et al. [37]	2013	Harari	239	> 95% of prescribed ARV drugs for the past 7 days.	Family or others	164/208	44/208	1.09
2	Mohammed et al. [38]	2015	Oromia	237	< 3 missed doses/day over study period	Community	175/197	22/197	3.41
3	Ketema et al. [36]	2015	Amhara	422	0 missed dose during the 30-day period prior to filling out the self-report.	Religious leaders and others	357/422	65/422	1.96
4	Alagaw et al. [33]	2013	SNNPR	357	> = 95% of prescribed drugs in past 7 days.	No specifics regarding disclosure	281/312	31/312	2.59
5	Tsega et al. [18]	2015	Amhara	351	< 3 missed doses over the entire time of therapy (Range: >6mo to < 3 yr)	Family	265/284	19/284	1.13
6	Gelan [35]	2010	SNNPR	277	Patients who reported an intake of 95% or more of the prescribed medication	Partner, family, relatives, friends and community	86/182	96/182	1.85
7	Asmare et al. [34]	2014	Amhara	377	< 3 missed doses per month	Family/Relatives	37/55	18/55	0.85

Note: all studies were cross-sectional

### Characteristics of included studies

The seven studies included in the review were all cross-sectional [18, 33–38]. They had a total of sample of 2260 adults living with HIV/AIDs in Ethiopia and were conducted from 2010 to 2015 in various regions of the country. The study sample sizes ranged from 233 to 422 participants [36, 38]. Of the seven studies included in the final analysis, three were conducted in the Amhara region [18, 34, 36] and two were conducted in the Southern Nations Nationalities and People (SNNPR) region [33, 35]. The remaining two studies were conducted in Oromia [38] and Harari regions [37] respectively.

The highest odds ratio (OR) for impact of sero-status disclosure on adherence was 3.41, reported for a study conducted in Oromia region [38]. The smallest odds ratio for sero-status disclosure (0.84) was reported in study conducted in Amhara region [34]. The latter study was the only study included which reported an OR below 1 (or a negative impact of disclosure on adherence). Although it went unmentioned by the study authors, lack of social support and its negative impact on both adherence and disclosure is well understood in the literature [39].

All included studies were conducted within a hospital or clinic environment. Geographically, they spanned three of the most populous regions in Ethiopia and contained a mix of both rural and urban populations.

### The effect of disclosure on adult antiretroviral therapy adherence

**Fig. 2:**
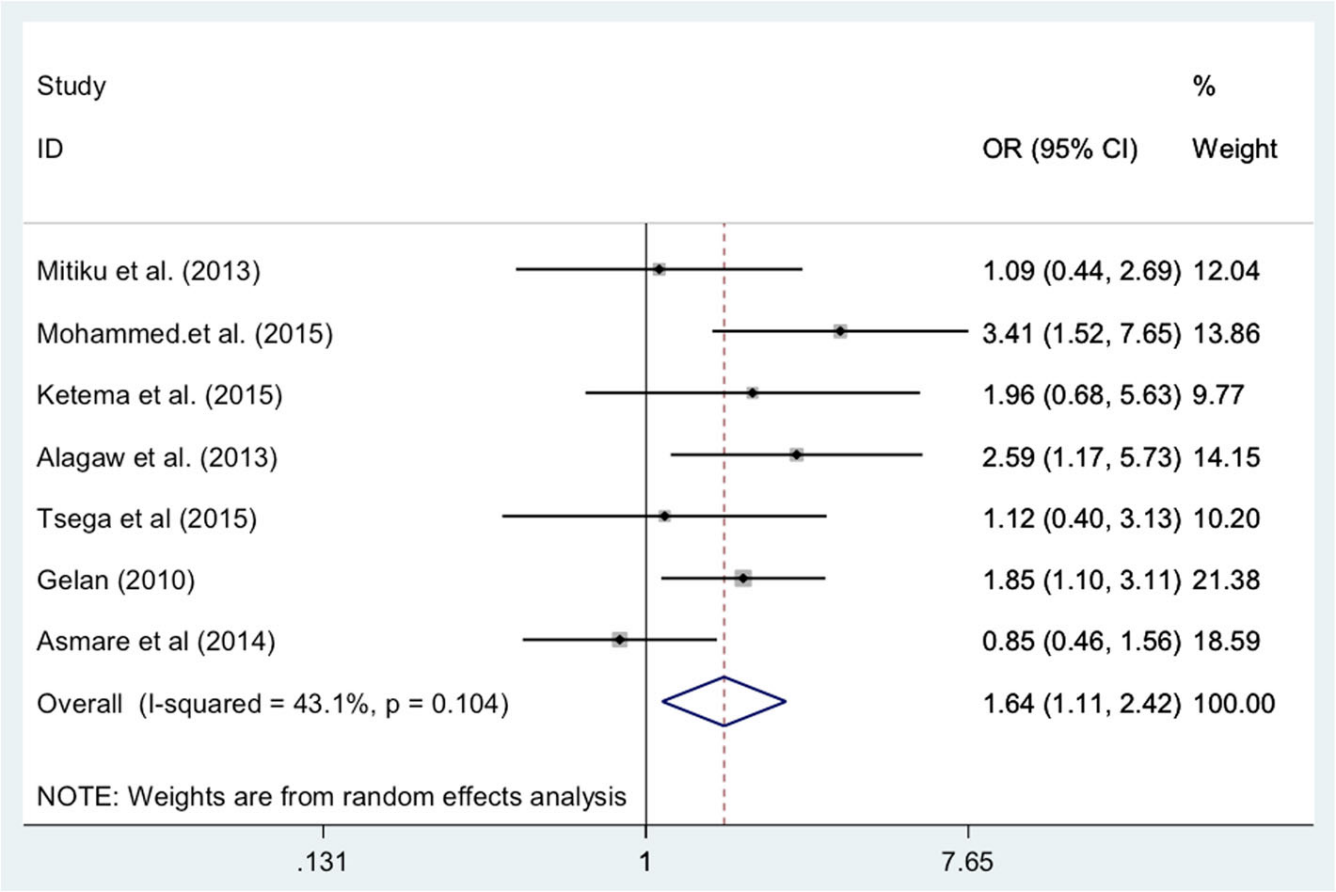
Forest plot of studies on the effect of disclosure on ART adherence among adult HIV patients, 2010–2015, Ethiopia

We found significant heterogeneity across studies (I^2^ = 42.9%, *p* = 0.105), which means that using a fixed effects model would have led to an unreliable estimate. Therefore, we used a random effects model to estimate the pooled effect of disclosure on the adherence reported by the seven studies with inverse variance. We found no evidence of publication bias after using a funnel plot of asymmetry and Egger’s test. Although visual examination of the funnel plot shows it to be asymmetric, Egger’s test of the intercept (B0) was 0.47 (95% CI: − 5.16, 6.11 p = 0. 0.28) (see Fig. 2).

Our meta-analysis found that sero-status disclosure had statistically significant effects on adherence to ART. Adults living with HIV who disclosed their sero-status were 1.64 times more likely to have good adherence compared to patients who did not disclose (OR: 1.64, 95% CI: 1.11, 2.42).

## Discussion

This systemic review and meta-analysis attempted to estimate the pooled effect of disclosure on adherence to ART among adults living with HIV in Ethiopia. Our analysis indicated that disclosure of sero-status had a statistically significant effect on adherence to ART. The odds of having good adherence among adults living with HIV who disclosed their sero-status were 64% higher than those who did not disclose their sero-status with (OR = 1.64 (95% CI: 1.11, 2.42)). The positive relationship between HIV disclosure status and adherence to ART in our analysis is consistent with the findings of primary studies and reviews conducted in high income settings, that found that HIV status disclosure was strongly correlated with ART drug adherence [40–42]. Our results are also in line with those from a systematic review of factors associated with adherence in sub-Saharan Africa, which found that disclosure among people living with HIV had a positive effect on ART adherence (OR = 3.46; 95% CI 2.04 to 5.89) [6].

Our estimated pooled odds ratios are more modest than the odds reported in the sub-Saharan Africa systematic review, reflecting perhaps the small number of studies that we had under review and the heterogeneity in the definitions of the independent and dependent variables. Another possible explanation may be that the positive impact of disclosure in Ethiopia is dampened (or reversed in the case of the study which found that it has negative impact) by a lack of social support in the country. Self-disclosure by itself does not improve adherence. Rather, one of the reasons that self-disclosure is important is that it helps PLWHA to ask for and receive crucial social support and, and, this in turn promotes better mental health and good ART adherence [39, 43]. In contrast, depression and poor social support have been show to negatively affect ART drug adherence [6, 44]. The relationship between social support and disclosure may be bi-directional in that poor social support may preclude disclosure if PLWHA do not feel safe in sharing their status. The literature and the studies included in our review suggest that the relationships between poor adherence and disclosure might be explained, in part, by inadequate social support leading to fear of taking drugs in front of family members; this is then leads patients to hide their sero-status from relatives, making it difficult to take medication at a prescribed time [45]. This suggests that programs to create supportive environments in which people feel safe to disclose, might have more of an impact on adherence than promoting disclosure on its own. Promoting support from key, highly respected parties such religious leaders, and providing counseling support for disclosure between couples and with relatives (rather than promoting disclosure to anyone) may be particularly important.

Our review adds to the literature by aggregating and highlighting articles germane to Ethiopian experiences. Ethiopia’s HIV epidemic is different than other epidemics in the region in that it is widely spread across the country, it is highly concentrated in certain geographic regions and in specific vulnerable populations. The country also has unique religious and socio-political structures in the Ethiopian Orthodox Church and the Gada System of the Oromo for example, which have the potential to influence the level of social support for PLWHA and their ability to disclose their HIV status. In addition, care-seeking behavior in Ethiopia is idiosyncratic, with its own traditions of alternative medicines and treatments such as the extensive use of holy water, among other things [36, 38, 46]. The use of these alternative treatments may also influence adherence and disclosure.

While many HIV prevention strategies are transferrable across settings, it is critical to have country-specific culturally relevant solutions for HIV prevention and treatment. Country-specific studies such as ours, aid in the development of these solutions. Our findings coupled with that from the literature, underscore the importance of active self-disclosure of sero-status and may serve as an important reminder for policy makers and program planners to strengthen existing strategies and explore novel or more aggressive strategies such as partner notification to support PLWHA to take this difficult step.

### Limitations

There are several limitations to this study. First our search yielded only a modest number of articles to review. Second, our findings were the pooled effect of many cross-sectional study designs, which are limited in their ability to draw upon causal inferences. The small number of studies included in the review and the patchiness of the data on respondent characteristics that they reported also limited our ability to conduct sub-group analyses of the studies in order to better explain the heterogeneity of the studies. Another important limitation is that the articles reviewed came primarily from smaller journals where the extent of peer review was difficult to assess, or from non-peer reviewed dissertations. In addition, although we followed standard PRISMA protocols, we must note that our study was not pre-registered. Finally, and most important, the studies that we reviewed lacked of standardized definitions for both good adherence and disclosure which may have increased the heterogeneity of our findings.

## Conclusion

Our systematic review and meta-analysis demonstrated that disclosure of HIV sero-status had a statistically significant positive effect on adherence to ART among adults living with HIV in Ethiopia. This suggests that Ethiopia’s national treatment and prevention programs should redouble efforts to encourage self-disclosure among people living with HIV/AIDS. Partner notification and partner disclosure support initiatives might be particularly helpful in this regard. Counseling guidelines for health professionals who work in HIV treatment and testing should emphasize discussing disclosure with patients. Future research should focus on documenting the ART support needs of priority vulnerable adult populations in Ethiopia especially sex workers, seasonal laborers, women, and men who have sex with men and determining the factors hinders them from disclosing and adhering to ART in order to identify solutions for managing their care compassionately and effectively.

## Data Availability

All data are available in the document.

## Appendix

### PubMed Advanced Search Terms

(((((adherence[All Fields] AND (“anti-retroviral agents”[Pharmacological Action] OR “anti-retroviral agents”[MeSH Terms] OR (“anti-retroviral”[All Fields] AND “agents”[All Fields]) OR “anti-retroviral agents”[All Fields] OR “antiretroviral”[All Fields])) AND (“association”[MeSH Terms] OR “association”[All Fields])) OR (“association”[MeSH Terms] OR “association”[All Fields])) AND (“disclosure”[MeSH Terms] OR “disclosure”[All Fields])) AND (“therapy”[Subheading] OR “therapy”[All Fields] OR “therapeutics”[MeSH Terms] OR “therapeutics”[All Fields])) AND (“ethiopia”[MeSH Terms] OR “ethiopia”[All Fields])

## Abbreviations

AN: Aynew Negesse
ART: Antiretroviral therapy
CI: Confidence interval
CT: Cheru Tesema
DA: Dessalegn Amare
DJ: Dube Jara
FW: Fasil Wagnew
GD: Getenet Dessie
HIV: Human immunodeficiency virus
HM: Henok Mulugeta
NOS: Newcastle-Ottawa Scale
OR: Odds ratio
PLWHA: People living with HIV/AIDS
SB: Sahai Burrowes
SR: Swati Rayasam
